# Age-dependent relationships between body mass index and mortality: Singapore longitudinal ageing study

**DOI:** 10.1371/journal.pone.0180818

**Published:** 2017-07-24

**Authors:** Tze Pin Ng, Aizhen Jin, Khuan Yew Chow, Liang Feng, Ma Shwe Zin Nyunt, Keng Bee Yap

**Affiliations:** 1 Gerontology Research Programme, Department of Psychological Medicine, National University of Singapore, Singapore; 2 National Disease Registry Office, Health Promotion Board, Singapore; 3 Department of Medicine, Alexandra Hospital, Singapore; University College London, UNITED KINGDOM

## Abstract

**Objectives:**

The relationship between body mass index (BMI) with mortality risk, in particular the BMI category associated with the lowest all-cause and CVD-and-stroke mortality and the BMI threshold for defining overweight or obesity in older persons is controversial. This study investigated the age-dependent associations of BMI categories with all-cause and cardiovascular disease (CVD) and stroke mortality.

**Method:**

Prospective cohort study (Singapore Longitudinal Ageing Studies) of older adults aged 55 and above, followed up from 2003 to 2011. *Participants* were 2605 Chinese with baseline BMI and other variables. *Outcome Measurement*: Mortality hazard ratios (HR) for all-cause and CVD and stroke mortality.

**Results:**

Overall, BMI showed a U-shaped relationship with all-cause and CVD and stroke mortality, being lowest at Normal Weight-II category (BMI 23.0–24.9 kg/m2). Most evidently among the middle-aged (55–64 years), all-cause mortality risks relative to Normal Weight-II were elevated for underweight (<BMI 18.5; HR = 4.92, p<0.0138), Normal Weight-I (BMI 18.5–22.9; HR = 3.41, p = 0.0149), and Overweight-Obese (BMI>30.0; HR = 4.05,p = 0.0423). Among the old (≥65 years), however, Overweight and Obese categories were not significantly associated with increased all-cause mortality (HR from 0.98 to 1.29), but Overweight-Obese was associated with increased CVD and stroke mortality (HR = 10.0, p = 0.0086).

**Conclusion:**

BMI showed a U-shaped relationship with mortality. Among older persons aged 65 and above, the overweight-or-obese category of BMI was not associated with excess all-cause mortality.

## Introduction

Body mass index (BMI) is known to be associated with a continuum of health risks, with many studies showing a U-shaped relationship. [1–7] Along this continuum, the appropriate BMI cut-off point to define overweight and obesity that is associated with increased mortality risk is controversial. In the 1980’s to 2000’s, the U.S. Dietary Guidelines have variously defined overweight using BMI of 24.9 to 27.1. In 1985, the U.S. National Institutes of Health (NIH) recommended that overweight be defined using BMI 27.8 for men and 27.3 for women. In the 1990s, the definitive guide set by both the NIH and World Health Organization (WHO) was BMI over 25 for overweight and BMI over 30 for obesity.

However, emerging evidence showed that Asian populations are at an increased risk of type 2 diabetes mellitus and cardiovascular diseases at lower BMI levels below 25 kg/m^2^ than their Western counterparts, and Asians with the same BMI as their Western counterparts have higher body fat percentages and greater abdominal and visceral fat deposition. [8] In 2004 the WHO recommended lowering the BMI cut-offs for Asian adults for overweight from 25 to 23 kg/m^2^ and for obesity from 30 to 27.5 kg/m^2^. [9] As there is evidently increased cardio-metabolic disease risk at an even lower BMI among South Asian populations, [10] India has adopted the lower BMI cut-off points of 23 kg/m^2^ for overweight and 25 kg/m^2^ for obesity. [11]

Large prospective cohort studies of Western populations variously indicated that the BMI category associated with the lowest all-causes mortality rate was at 22.5 to 25.0 kg/m^2^, [12] or 20.0 to 25.0 kg/m^2^, [13] whereas other studies [14–18] showed that BMI category 25-<30 typically associated with overweight, was associated with lower all-causes mortality, compared to normal weight (BMI of 18.5-<25). Reports of mortality risks associated with BMI among Asians were also mixed. [1,2,4,5,7,19–21] The relationship between BMI and all-cause and cardiovascular disease (CVD) and stroke mortality may depend on age, being attenuated or reversed in older age groups. [2, 22–24] Divergent findings may also be due to distortions produced by weight loss because of pre-existing disease (so-called ‘reverse’ causality). It is pertinent to note that the desired level of BMI recommended in clinical guidelines on the management of overweight and obesity in adults were primarily based on studies in young and middle-aged cohorts, [25] and may not be relevant in older persons.

The objectives of this study were to investigate the associations of BMI categories with all-causes and CVD and stroke mortality in a population cohort of middle-aged and older Chinese men and women in Singapore. We used the International Classification recommended by WHO of Underweight (<18.5), Normal weight-I (18.5–22.9), Normal weight-II (23.0–24.99), Overweight Pre-obese-I (25.0–27.49), Overweight-Pre-obese-II (27.5–29.99), Overweight-Obese Class I, II, III (≥30.0), and sought in particular to determine the BMI category associated with the lowest all-cause and CVD-and-stroke mortality, and whether the mortality risk associated with over-weight and obesity was age-dependent for younger middle-aged (55 to 64 years) and older (65 and over) individuals.

## Method

### Study design

We conducted a prospective follow up study of mortality in a cohort of 2808 participants in the Singapore Longitudinal Ageing Study (SLAS) from September 2003 to December 2011. We excluded those who reported recent loss of weight of 5 kg or more in the last six months (N = 104), and those with missing data on BMI and other variables. The final sample comprised 2604 Chinese participants with BMI data in this study.

### Study population

Between September 2003 and December 2005, a whole population of older adults aged 55 years and above who were Singaporean residents in contiguous precincts in the South East region of Singapore were identified from a door-to-door census and invited to participate in the Singapore Longitudinal Ageing Study (SLAS). The response rate was 78.2%. All participants provided written informed consent. The study was approved by National University of Singapore Institutional Review Board. Full details of the survey procedures and baseline variables and data collection are described in previous publications. [26]. Baseline information on demographic and socioeconomic status, medical history, physical activity, and smoking and alcohol history were collected from face-to-face interviews conducted by trained nurses using structured questionnaires at the participants’ home, and physical examination and testing were conducted at a local study site.

### Measures

Body mass index (weight divided by height-squared (kg/m^2^) was determined from measurements of the participant’s weight (in kilogram) and height (in metres). Based on BMI, participants were classified as Underweight (<18.5), Normal weight-I(18.5–22.9), Normal weight-II (23.0–24.99), Overweight Pre-obese-I (25.0–27.49), Overweight-Pre-obese-II (27.5–29.99), Overweight-Obese Class I, II, III (≥30.0), using the International Classification recommended by WHO.

### Covariates

Baseline data on sociodemographic and lifestyle variables were collected and categorized for education (>6 years, < = 6 years), smoking status (non-smoker, past smoker, current smoker), alcohol intake (≥1 drinks daily). Physical activity was assessed using questions on the frequency (0 = never or less than once a month; 1 = sometimes, i.e. once a month or more but less than once a week; 2 = often, i.e. at least once a week) of participation in brisk walking, physical workout routines, sports activities, and other physical recreational activities (e.g. taiji and qigong) and summed scores of physical activity score were categorized (0–1, 2–3, 4–8) for analyses.

### Mortality follow up

Information on date and cause of death of each study participant during follow up from baseline up to 31 December 2011 was determined using their unique National Registration Identity Card (NRIC) number for computerized record linkage with the National Death Registry through the National Disease Registry Office (NDRO) of the Ministry of Health.

### Statistical analyses

Comparison of baseline characteristics between study participants by BMI categories were performed with significance testing using t-test for continuous variables and χ^2^ for categorical variables. Univariate and multivariate Cox proportionate hazard regression analyses were used to estimate hazard ratio (HR) with 95% confidence intervals (95% C.I.) of all-causes and CVD and stroke mortality risks associated with BMI categories. Age (continuous), sex (men versus women), education (≤6 years versus >6 years), smoking (never, past and current smoking), alcohol intake (>1 drink daily: yes versus no), and physical activity level (scores of 0–1, 2–3, 4–8) were included as adjustment covariates in the Cox models. In stratified analyses, we assessed whether the association between BMI and the risk of death varied according to age group: younger middle-aged (55–64 years and older-aged (≥65 years).

## Results

Table 1 shows the demographic and health risk profiles of the study participants by BMI categories. Among the total of 2604 participants, 6.1% had BMI<18.5 (Underweight), 17.9% had BMI: 25.0–27.49 (Overweight Pre-obese-I), 8.6% had BMI: 27.5–29.99 (Overweight-Pre-obese-II) and 4.6% had BMI: ≥30.0 (Overweight Obese Class I, II, and III). Participants who were underweight were significantly older, and more likely to be current smokers at the start of the follow up.

**Table 1:** Baseline characteristics by BMI status.

	<18.5 Underweight	18.5–22.9 Normal-I	23.0–24.99 Normal-II	25.0–27.49 Overweight Pre Obese-I	27.5–29.99 Overweight Pre obese-II	≥30.0 Overweight, Obese Class I, II, III	P
N	158	1039	595	467	224	121	0.0049
Age (mean, SD)	67.7 (8.5)	65.9 (8.0)	65.9 (7.1)	66.5 (7.5)	65.5 (6.9)	64.4 (7.5)	0.0017
55–64	63 (40.1)	526 (50.8)	295 (49.6)	225 (48.4)	113 (50.7)	70 (58.3)	
65–84	87 (55.1)	484 (46.6)	295 (49.6)	235 (50.3)	109 (48.7)	51 (42.2)	
85-	8 (5.1)	29 (2.8)	5 (0.8)	7 (1.5)	2 (0.9)	0	
Female	95 (60.1)	690 (66.4)	356 (59.8)	271 (58.0)	154 (68.8)	76 (62.8)	0.0057
Male	63 (39.9)	349 (33.6)	239 (40.2)	196 (42.0)	70 (31.3)	45 (37.2)	
Education: >6 years	67 (42.4)	538 (51.8)	289 (48.6)	205 (43.9)	98 (43.8)	53 (43.8)	0.0181
< = 6 years	91 (57.6)	501 (48.2)	306 (51.4)	262 (56.1)	126 (56.3)	68 (56.2)	
Smoking: Non-smoker	123 (77.9)	883 (85.1)	490 (82.6)	382 (82.0)	190 (84.8)	102 (84.3)	0.0099
Past smoker	15 (9.5)	87 (8.4)	67 (11.3)	59 (12.7)	26 (11.6)	14 (11.6)	
Current smoker	20 (12.7)	68 (6.6)	36 (6.1)	25 (5.4)	8 (3.6)	5 (4.1)	
Alcohol: >1 drinks daily	6 (3.8)	19 (1.8)	11 (1.9)	9 (1.9)	0	2 (1.7)	0.1703
Physical activity score:							
0–1	51 (32.9)	297 (29.0)	168 (28.5)	139 (30.0)	82 (37.1)	40 (33.6)	0.0734
2–3	56 (36.1)	329 (32.1)	183 (31.2)	150 (32.4)	73 (33.0)	45 (37/8)	
4–8	48 (31.0)	398 (38.9)	238 (40.4)	174 (37.6)	66 (29.9)	34 (28.6)	

There were a total of 224 deaths among the 2605 study participants with 8,440 person-years of follow up. The number of deaths from CVD and stroke was 38 (17%) and from non-CVD and stroke was 186 (83%).

### BMI categories and all-cause mortality

**Fig 1:**
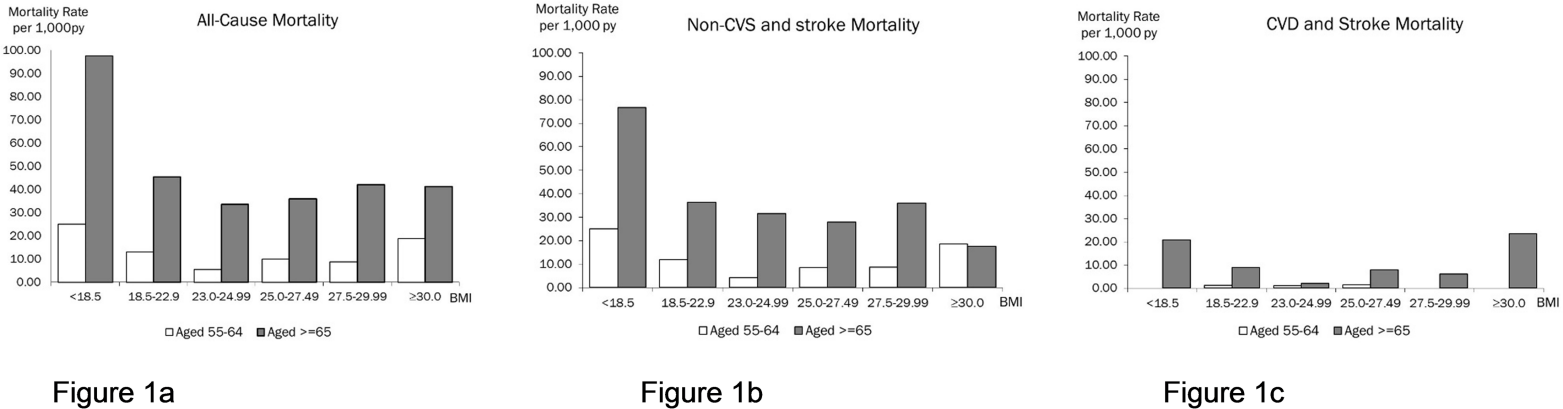
Mortality rates for All Causes, non-CVS and stroke and CVD and stroke by BMI categories among middle-aged (55–64 years) and old-aged (> = 65 years) participants.

**Fig 2:**
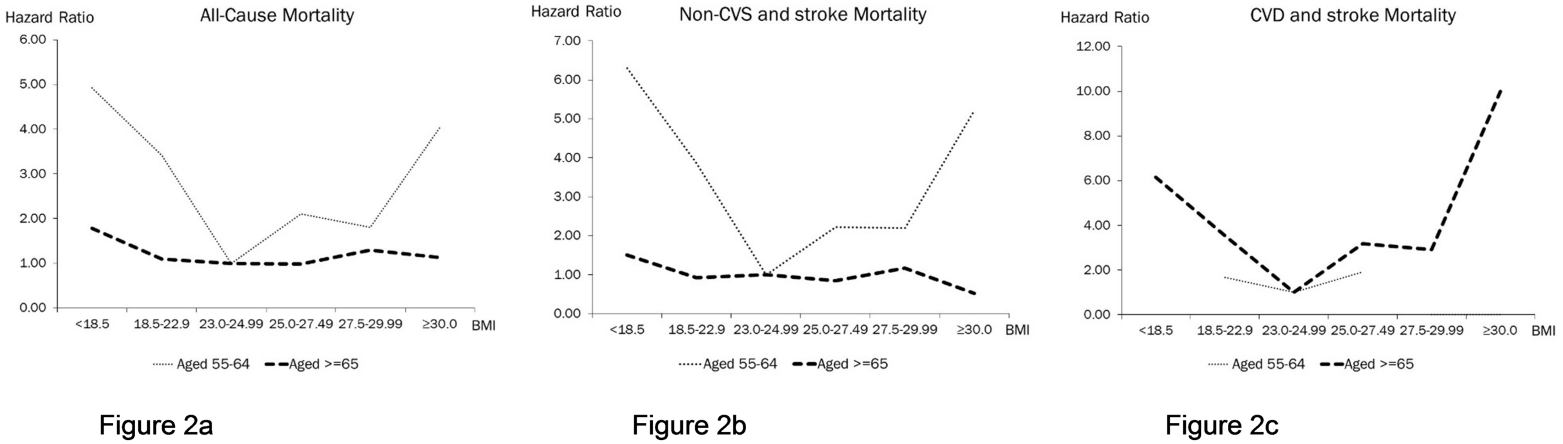
Mortality hazard ratios for All causes, Non-CVS and stroke and CVD and stroke by BMI categories among middle-aged (55–64) and old-aged (> = 65 years) participants.

The rates and relative risks for all-cause mortality across all categories of BMI showed a U-shaped relationship, being lowest at Normal Weight-II category (BMI 23.0–24.9 kg/m2). (Table 2; Figs 1a and 2a) This was clearly evident among the middle-aged (55–64 years), among whom all-causes mortality risks relative to Normal Weight-II were higher for Underweight (HR = 4.92, p<0.0138), Normal Weight-I (HR = 3.41, p = 0.0149) and Overweight-Obese (HR = 4.05, p = 0.0423).

**Table 2:** Associations of BMI with total and cardiovascular mortality by age strata.

	Total Mortality			
BMI categories	Pop/deaths	/1000 p-y	Adj HR	(95% C.I.)
Whole cohort				
<18.5 (Underweight)	158/33	67.7	2.06	(1.27–3.35)
18.5–22.9 (Normal weight)	1039/92	29.1	1.32	(0.90–1.95)
23.0–24.99(Normal-weight-II)	595/37	19.7	1.00	
25.0–27.49 (Overweight Pre-obese-I)	467/34	23.5	1.12	(0.70–1.78)
27.5–29.99 (Overweight-Pre-obese-II)	224/17	25.1	1.34	(0.75–2.38)
≥30.0 (Overweight Obese Class I, II, III)	121/11	28.6	1.52	(0.77–2.98)
Middle-aged cohort, 55–64 years				
<18.5 (Underweight)	63/5	25.0	4.92	(1.38–17.47)
18.5–22.9 (Normal weight)	526/21	13.2	3.41	(1.27–9.17)
23.0–24.99(Normal-weight-II)	295/5	5.4	1.00	
25.0–27.49 (Overweight Pre-obese-I)	225/7	10.0	2.10	(0.66–6.65)
27.5–29.99 (Overweight-Pre-obese-II)	113/3	8.7	1.80	(0.42–7.67)
≥30.0 (Overweight Obese Class I, II, III)	70/4	18.7	4.05	(1.05–15.65)
Old cohort, 65+				
<18.5 (Underweight)	95/28	97.6	1.78	(1.05–3.02)
18.5–22.9 (Normal weight)	513/71	45.3	1.09	(0.71–1.66)
23.0–24.99(Normal-weight-II)	300/32	33.6	1.00	
25.0–27.49 (Overweight Pre-obese-I)	242/27	35.9	0.98	(0.58–1.65)
27.5–29.99 (Overweight-Pre-obese-II)	111/14	42.0	1.29	(0.68–2.43)
≥30.0 (Overweight Obese Class I, II, III)	51/7	41.2	1.13	(0.50–2.59)

Among the older participants (≥65 years), however, only Underweight was associated with higher all-cause mortality (HR = 1.78, p = 0.0336), whereas Overweight and Obese categories were not significantly associated with increased all-causes mortality (HR from 0.98 to 1.29).

### BMI categories and CVS and stroke mortality

There were small counts of death, and no meaningful relationships were discernible for deaths from CVD and stroke among the young middle-aged. However, among the older participants aged 65 and above, both Underweight (HR = 6.15, p = 0.0299) and Overweight-Obese (HR = 10.0, p = 0.0086) were significantly associated with markedly increased CVD and stroke mortality (Figs 1b, 1c, 2b and 2c).

## Discussion

Our study confirms previous findings describing the U-shaped relationship of BMI with all-causes mortality. [1–7] In this population of middle-aged and older Asians, the BMI category associated with the lowest all-cause mortality was 23 to 24.99 kg/m^2^ (Normal Weight II). This is consistent with meta-analyses indicating that the all-cause mortality rate was lowest at 22.5 to 25.0 kg/m^2^. [12]

In agreement with previous studies, [6–7, 12] we also observed an age-dependency of associations of BMI with mortality risk in that the relative risk of death associated with BMI was higher among middle-aged persons (aged 55–64) and decreased among older persons (> = 65). For example, the HR of total mortality associated with being underweight was 4.92 in the middle-aged group and 1.78 in the older age group. The HR of total mortality associated with being overweight-obese (BMI> = 30) was 4.05 in the middle-aged group and 1.13 in the older age group

We thus observed that among older persons aged 65 and above, being overweight or obese compared to normal weight was not significantly associated with increased all-cause mortality risks. This agrees with previous studies involving older populations. [2, 15–19] Although among older persons, being overweight or obese was significantly associated with markedly increased CVD and stroke mortality (HR = 10.0, p = 0.0086), CVD and stroke mortality however comprised less than a quarter of deaths among the older participants. Thus the lack of association of overweight-obesity with increased total mortality among the older participants was due to lower mortality predominantly from non-vascular causes of death (cancer, pneumonia, injury, COPD and others).

There are more than a few explanations for the lowered mortality associated with being overweight and obese among older persons. One explanation is the limitation of BMI as an indicator of body fat, as older persons tend to have more body fat at the same BMI as younger adults, with greater BMI possibly reflecting relatively greater fat-free mass, rather than greater body fat. Another explanation is survival bias. Because obese persons were more likely to die earlier at younger ages, those who survived into old age were selectively healthier, commensurate with recent observations of a population subgroup of obese people who are ‘metabolically healthy’. [27–29]. Another explanation is confounding due to disease-associated unintentional weight loss prior to death among obese individuals. This may result in shifting misclassification of obese individuals into the categories of low or normal BMI category. This explanation for our observed association is considered to be less likely as we excluded those who reported recent loss of weight of 5 kg or more in the last six months from various causes. Another possible explanation is the protective effect of adiposity against fatal health outcomes in late life. In opposition to the increased risk of death of persistent weight gain from early life, weight gain late in life may have possible beneficial effects, for example, through its association with increased bone mineral density (BMD), decreased osteoporosis, falls and hip fracture, and decreased mortality risks. [30–32] Elderly patients with chronic heart failure and end-stage renal failure with higher BMI are also known to have all-cause mortality that were lower compared to their non-obese counterparts, [33–36] a phenomenon that is widely referred to as the “obesity paradox”. [37–39]

In this study, underweight was found to be associated with increased all-cause mortality both among middle-aged and older individuals, including increased CVD-and-stroke mortality among older participants. This is expected, and is in agreement with many studies. Among older people, being under-weight largely reflects decreased muscle mass and strength, and is related to an increased risk of sarcopenia and frailty-associated functional limitation and mortality. [40–42] Because we have excluded those who reported significant weight loss prior to follow up, we believe that this reflects the increased risk from leanness, more than the confounding association due to pre-existing disease.

A limitation in this study is the relatively small number of deaths observed in the middle-aged group and for cardiovascular disease and stroke. In particular, there were no deaths due to CVD and stroke recorded in the middle-aged group, and the small number of deaths due this cause of death among the older group could contribute to the very high hazard ratio estimate associated with obesity. Because the estimates of odds ratio were imprecise, the age-differential in vascular disease-related mortality associated with BMI should therefore be interpreted with caution. The limitation of BMI measure to estimate adiposity is widely acknowledged in the literature. Further studies using waist circumference and other metabolic indicators of body composition should provide a much needed clearer understanding of the specific health risks associated with muscle mass and fat mass.

From a clinical standpoint, the controversy over the association between high BMI and lowered mortality in older adults may lead to misinterpretations or confusions regarding the desired level of BMI in older persons. Certainly, it should not be interpreted to mean that obesity conveys a lower mortality risk in older persons compared to younger and middle-aged persons, because the absolute mortality risk associated with increased BMI is still higher in older persons because of the marked increase in mortality with advancing age, as shown in the Table 3 and Fig 1a. However, the desired level of BMI recommended in clinical guidelines on the management of overweight and obesity in adults were primarily based on studies in young and middle-aged cohorts, [25] and may not be relevant in older persons. Among older adults, the health risk of under-weight or weight loss in older persons is a concern, largely reflective of the health risks of sarcopenia and frailty. Certainly, older persons who are obese with metabolic complications may benefit from weight-loss therapy if muscle and bone losses are minimized. [43] Studies have demonstrated the feasibility of exercise-induced reduction in waist circumference and abdominal fat without a corresponding reduction in BMI, [42–44] but more studies are needed.

**Table 3:** Associations of BMI with total and cardiovascular mortality by age strata.

	Non-Cardiovascular and Stroke Mortality				Cardiovascular and Stroke Mortality			
BMI categories	Pop/deaths	/1000 p-y	Adj HR	(95% C.I.)	Pop/deaths	/1000 p-y	Adj HR	(95% C.I.)
Whole cohort								
<18.5 (Underweight)	158/27	55.4	1.89	(1.12–3.20)	158/6	12.3	3.99	(0.96–16.58)
18.5–22.9 (Normal weight)	1039/76	24.0	1.20	(0.80–1.81)	1039/16	5.1	2.73	(0.79–9.44)
23.0–24.99(Normal-weight-II)	595/34	18.1	1	(referent)	595/3	1.6	1	(referent)
25.0–27.49 (Overweight Pre-obese-I)	467/27	18.6	0.99	(0.60–1.65)	467/7	4.8	2.48	(0.63–9.70)
27.5–29.99 (Overweight-Pre-obese-II)	224/15	22.1	1.28	(0.70–2.36)	224/2	2.9	1.89	(0.31–11.34)
≥30.0 (Overweight Obese Class I, II, III)	121/7	18.2	1.05	(0.47–2.39)	121/4	10.4	6.62	(1.45–30.11)
Middle-aged cohort, 55–64 years								
<18.5 (Underweight)	63/5	25.0	6.30	(1.65–24.07)	63/0	NA	NA	NA
18.5–22.9 (Normal weight)	526/19	11.9	3.85	(1.29–11.49)	526/2	1.3	1.67	(0.14–20.48)
23.0–24.99(Normal-weight-II)	295/4	4.3	1	(referent)	295/1	1.1	1	(referent)
25.0–27.49 (Overweight Pre-obese-I)	225/6	8.6	2.23	(0.62–7.96)	225/1	1.4	1.92	(0.11–34.76)
27.5–29.99 (Overweight-Pre-obese-II)	113/3	8.7	2.20	(0.48–10.03)	113/0	NA	NA	NA
≥30.0 (Overweight Obese Class I, II, III)	70/4	18.7	5.22	(1.26–21.60)	70/0	NA	NA	NA
Old cohort, 65+								
<18.5 (Underweight)	95/22	76.7	1.50	(0.84–2.67)	95/6	20.9	6.15	(1.19–31.68)
18.5–22.9 (Normal weight)	513/57	36.3	0.93	(0.59–1.46)	513/14	8.9	3.53	(0.79–15.71)
23.0–24.99(Normal-weight-II)	300/30	31.5	1	(referent)	300/2	2.1	1	(referent)
25.0–27.49 (Overweight Pre-obese-I)	242/21	28.0	0.84	(0.48–1.47)	242/6	8.0	3.17	(0.63–15.93)
27.5–29.99 (Overweight-Pre-obese-II)	111/12	36.0	1.17	(0.60–2.31)	111/2	6.0	2.91	(0.41–20.72)
≥30.0 (Overweight Obese Class I, II, III)	51/3	17.6	0.52	(0.16–1.72)	51/4	23.5	10.0	(1.80–55.67)

Covariates in in Cox regression model are: age (continuous), sex (men, women), education (≤6, >6 years), smoking, alcohol, physical activities
